# Effectiveness of Structural–Strategic Family Therapy in the Treatment of Adolescents with Mental Health Problems and Their Families

**DOI:** 10.3390/ijerph16071255

**Published:** 2019-04-08

**Authors:** Lucía Jiménez, Victoria Hidalgo, Sofía Baena, Antonio León, Bárbara Lorence

**Affiliations:** 1 Faculty of Psychology, University of Seville, Camilo José Cela s/n, 41018 Seville, Spain; luciajimenez@us.es (L.J.); mbaena3@us.es (S.B.); bll@us.es (B.L.); 2 Child and Adolescent Mental Health Unit, Virgen Macarena Hospital, C/ Dr. Fedriani, 3, 41009 Seville, Spain

**Keywords:** family therapy, effectiveness, strategic therapy, structural therapy, family functioning, parental competence, parenting alliance, behavior problems, mental health

## Abstract

Mental health problems during adolescence constitute a major public health concern today for both families and stakeholders. Accordingly, different family-based interventions have emerged as an effective treatment for adolescents with certain disorders. Specifically, there is evidence of the effectiveness of concrete approaches of systemic family therapy on the symptoms of adolescents and family functioning in general. However, few studies have examined the effectiveness of other relevant approaches, such as structural and strategic family therapy, incorporating parent–child or parental dyadic measurement. The purpose of this study was to test the effectiveness of a structural–strategic family therapy with adolescents involved in mental health services and their families. For this purpose, 41 parents and adolescents who participated in this treatment were interviewed at pre-test and post-test, providing information on adolescent behavior problems, parental sense of competence, parental practices, parenting alliance, and family functioning. Regardless of participants’ gender, adolescents exhibited fewer internalizing and externalizing problems after the treatment. Parents reported higher family cohesion, higher satisfaction and perceived efficacy as a parent, and healthier parental practices (less authoritarian and permissive practices, as well as more authoritative ones). An interaction effect between parenting alliance and gender was found, with more favorable results for the mothers. In conclusion, this paper provides evidence of the usefulness of structural–strategic family therapy for improving family, dyadic, and individual facets in families with adolescents exhibiting mental health problems.

## 1. Introduction

Mental health problems during adolescence constitute a major public health concern today for both families and stakeholders [1,2]. Epidemiological studies show that mental health issues are the first nonfatal cause of illness [3], are in the top five causes of death among adolescents [4], and represent 16% of the global health-related burden in young people [4,5]. In addition, mental health problems during adolescence are an important predictor of socialization difficulties and absenteeism at this developmental stage, as well as one of the most significant predictors of adjustment problems and mental disorders in adulthood [6,7,8]. In order to address these pressing issues, it is essential to have effective intervention and prevention strategies that meet the specific needs of adolescents with mental health problems. 

Adolescence is a challenging transitional period for both children and families. It is a developmental stage characterized by normative physical, social, and psychological changes [9], some of which may be identified as potentially stressful among this population [10]. Psychosocial stress in adolescents can be accentuated by the presence of stressful or adverse life events (as maltreatment and violence, loss events, intrafamilial problems, school and interpersonal problems) that are associated with severe negative outcomes [11]. Although there are important inter-individual differences, the current homogenization of adolescents’ daily experiences has contributed to the observation of fewer cross-cultural and gender differences during this stage [12]. Some of the normative developmental tasks that adolescents need to undertake for a healthy development are the search for autonomy, identity, and independence [9]. For families, this is a period characterized by the readjustment of family roles and norms, along with an increase in family conflicts [9,13,14]. Families face the challenge of adjusting to these new demands and needs while trying to conserve family unity [9,13,14]. The inability to adjust to these new demands, together with inflexibility within the family over the negotiation of new norms and different solutions, are often related to mental health problems. Families with an adolescent with mental health problems have additional needs, demands, and difficulties stemming from the mental disorder [15]. Parents often face challenging behaviors and conflictive situations, having to manage symptoms and coordinate and engage with different service systems [16,17]. As they struggle to deal with these additional demands, parents often find their skills coming into question, and this can be accompanied by feelings of low competence, frustration, and powerlessness, together with increased isolation and contraction in their social network [15,18].

There has been a proliferation of family-oriented and family-based interventions with adolescents with mental health difficulties; some of these are considered as evidence-based practices in the treatment of children and adolescents with certain disorders [19,20]. Previous research indicates that the incorporation of family members or family elements in therapy is either directly or indirectly an effective component of interventions that target adolescents with mental health problems [21,22,23]. On one hand, direct approaches (e.g., family-centered behavioral management or family therapy) involve a more immediate engagement with the family and usually include specific objectives that target families or family members. On the other hand, indirect approaches (e.g., psychodynamic therapy or cognitive–behavioral therapy) incorporate the family context through reviews or reports, using them as informants at some point and by keeping the family elements in mind while intervening [22]. In sum, under the “family-based interventions” umbrella term, there are a wide range of qualitatively different interventions and approaches. The most widely used family-based interventions include psychoeducational approaches [24], behavioral interventions, and systemic family therapy [25]. The goal of the present study was to evaluate the efficacy of specific systemic family therapy approaches in families with an adolescent presenting a mental health problem.

From a systemic perspective, family is defined as a transactional system, where difficulties in any member have an influence on every other member and on the whole family as a unit. In turn, family processes have an impact on every individual member, as well as on the different relationships embedded within the family context [26]. This perspective shifts away from a linear consideration of family processes by recognizing the multiple recursive influences that shape family relationships and family functioning, perceiving it as an ongoing process throughout the life cycle [27]. Systemic family therapy has been shown to be an efficacious intervention for families and adolescents with a wide range of mental health problems, such as drug use [19,28,29,30,31,32]), eating disorders [29,30] and both internalizing and externalizing disorders [19,29,30,31,33,34,35,36]. Despite these advances, most of the literature has focused on either systemic family therapy as a whole, without taking into account the different approaches embedded within this framework, or on the effectiveness of more manualized approaches, such as multisystemic family therapy (e.g., [37]) or functional family therapy (e.g., [34,38]). Few studies have examined the effectiveness of more classical and widely used approaches, such as structural and strategic family therapy [39]. Hence, more research is needed to be able to draw more definite conclusions regarding the use of these types of family therapy approaches.

Structural family therapy is one of the dominant approaches in systemic family intervention, originally created by Minuchin [40]. The focus of this approach is on achieving a healthy hierarchical family organization, where there are different subsystems with their limits and boundaries [27,41]. According to this approach, the difficulties expressed by the adolescent are a reflection of: (1) A family structural imbalance; (2) a dysfunctional hierarchy within the family system, often characterized by difficulties in establishing boundaries between the parental and the child subsystem; and (3) a maladaptive reaction to changing demands [27]. Therefore, the intervention focuses on reinforcing the parental subsystem, highlighting the need to present a “united front”, and clearly differentiating it from the parent–child subsystem [25,27,41,42]. It also emphasizes the need to adjust the rigidity of the limits and the relationship between subsystems according to the moment of the life cycle [42]. During adolescence, while authority still relies on the parental subsystem, the way it is exerted cannot be the same as in previous developmental stages, and the limits between the subsystems, while remaining clear, have to be more flexible [25,27,42]. Although the core elements of this approach are well established and widely used among the clinical community [30,43], few studies have addressed the effectiveness of this approach for adolescents with mental health problems [39,44]. 

Strategic family therapy is purely embedded within the systemic model and has a more directive impression [25,45]. From this approach, the symptom is considered as serving a function to the family, as well as reflecting a difficulty of the family to solve a problem [25,27,45]. According to the strategic approach, when faced with a problem, families adopt solutions that have been useful to them in the past. However, symptoms such as behavioral or emotional difficulties or an increase in conflicts emerge for which those solutions are no longer valid, and the family is unable to find and effectively use alternative ones; thus, they become stuck in a symptom-maintaining sequence [27]. The objective of this therapy is for the family to initiate actions and solutions that are different to the ones previously attempted [27,45]. There is extensive evidence about the effectiveness of the brief–strategic family therapy approach, which is a manualized and specific variant of the strategic approach, with different populations [46], including adolescents with mental health problems (e.g., [32,47,48]). Though structural and strategic family therapy are conceptually two different approaches within the systemic framework, they share certain core elements, and it is not rare to use them conjointly. Some illustrative examples are brief–strategic family therapy and multisystemic therapy, both of which incorporate representative elements from both approaches.

In general, literature has shown that systemic family therapy has a significant impact by reducing internalizing and externalizing symptoms of adolescents, as well as improving overall family functioning [35,36]. However, in spite of the evidence indicating gender differences in adjustment problems, especially in internalizing symptoms, most available studies have not taken into account the adolescent’s gender when examining the impact of these interventions [49]. In addition, most studies have focused on individual outcomes or on family functioning as a whole, rather than incorporating parent–child dyadic measures or parental dyadic measures. Research has shown that some of these dyadic dimensions play an important role in families with adolescents with mental health problems; they should therefore be incorporated in effectiveness evaluations. More specifically, coercive and permissive parenting practices [50,51,52] have generally been considered as two of the most important predictors of internalizing and externalizing problems. Other parenting dimensions linked to child psychopathology include: Low sense of parental competence, defined as the perception parents have of their own performance as parents [52,53,54], and high levels of interparental conflict [55]. As a result, parental practices, sense of parental competence, and parenting alliance constitute intervention targets and should be included in effectiveness evaluations.

For some of these dimensions, the studies available highlight the need to control gender differences. Specifically, there is evidence of important differences in parenting practices between mothers and fathers, with mothers scoring higher in communication and control dimensions [56,57,58]. In addition, there is evidence of gender differences in the perception of parenting alliance and co-parenting; more specifically, in parental support and involvement dimensions. Thus, mothers are more likely to be involved in parental decision-making processes than fathers but also feel less supported in their parental role [59].

In this framework, the goal of this study was to evaluate the effectiveness of structural–strategic family therapy on different individual, dyadic, and family dimensions in families with an adolescent with a mental health problem; to do so, we conducted a comprehensive analysis and incorporated a gender perspective. According to previous evidence on systemic family therapy, we expected a reduction of internalizing and externalizing symptoms of adolescents, as well as an improvement in family functioning. Due to their role in child psychopathology, a reduction of coercive and permissive parenting practices as well as an increase in sense of parental competence and parenting alliance were hypothesized. Because of an absence of previous studies, we did not have expectations regarding the adolescent’s gender, although higher improvements in mothers were expected in comparison to fathers.

## 2. Materials and Methods

### 2.1. Study Design

This study was part of a wider research project assessing the effectiveness of a structural–strategic family therapy (SSFT) initiative run by mental health services in Southern Spain (Andalusia) for families with an adolescent with a mental health problem. This initiative combined the theoretical principles and techniques of structural and strategic family therapy in order to reduce the adolescent’s mental behavior problems and improve family relationships. The family therapy sessions initially focused on establishing a therapeutic alliance with all members of the family, providing them with a safe, nonjudging space where all of them felt understood. Afterwards, the objectives of the sessions were to set clear boundaries between the subsystems, to strengthen the parental subsystem encouraging joint decision-making and teamwork, to highlight and balance parental authority with the increasing need for autonomy from the adolescent, and to reframe the relationships within the family system. Both the referred adolescent with a mental health diagnosis and his/her parents participated in SSFT; any other significant family members were also asked to attend. The intervention was led by two therapists trained in structural and strategic family therapy (a clinical psychologist and a psychiatrist). On average, the treatment consisted of a one-hour session each month over a period of approximately 10 months [60].

For the purpose of the evaluation, a quasi-experimental design was followed, including a pre-test versus post-test evaluation of the participants of an experimental group (EG). This EG consisted of the population of families receiving the SSFT intervention during the study (i.e., between 2009 and 2012).

### 2.2. Participants

The sample consisted of 41 participants (51.22% mothers, 48.78% fathers), whose adolescent children had been referred to mental health services in the South of Spain. The children’s ages ranged between 10 and 17 (M = 14.12, SD = 1.79), and there was a higher percentage of girls (73.17% girls and 26.83% boys). Most families were two-parent (90.24%), with nearly all of them having four members (M = 3.82, SD = 0.85) and an average of two children (M = 1.80, SD = 0.51).

Following ICD-10 criteria, behavioral disorders were the most common diagnoses (31.71%), followed by anxiety (29.27%), mood (17.07%), and eating disorders (17.07%). Other less frequent diagnoses included personality disorders (9.76%), psychotic disorders (9.76%), and pervasive developmental disorders (4.88%). Approximately 20% of adolescents with one type of disorder met the criteria for another class of disorder (19.51%), with half of the comorbidities between behavioral and anxiety disorders (9.76%) and the other half between anxiety and mood disorders (9.75%).

### 2.3. Measures

The study followed a multi-informant approach, collecting information from practitioners, caregivers, and target adolescents. In this paper, information provided by practitioners and caregivers is included. Practitioners provided information about adolescent and family sociodemographic profiles. Caregivers informed about the target adolescent behavior, as well as about their parental sense of competence, parental practices, perceived parenting alliance, and perceived family functioning. These measures are described below.

*Sociodemographic profile*: We compiled an ad-hoc questionnaire to collect sociodemographic information about the target adolescent’s age and gender (by measuring sex) and the family structure (one/two-parent structure) and composition (number of family members and children at home). 

*Child behavior checklist for ages 6–18* [61]: This inventory provides information on child and adolescent behaviors from the perspective of caregivers. It measures both positive competences and problem behaviors (internalizing and externalizing). A compilation of 113 items (ranging from 0 = *not true* to 2 = *very true or often true*) measures internalizing (withdrawn/depressed, somatic complaints, and anxiety/depression) and externalizing problems (rule-breaking and aggressive behavior). Cronbach’s alpha coefficients were α = 0.85 for internalizing problems and α = 0.89 for externalizing problems. Higher scores indicate greater behavior problems. Mean scores were computed.

*Parental sense of competence* [62]: This scale explores perceived competence as a parent. It consists of 16 items with responses on a six-point scale. Two subscales can be computed, measuring efficacy and satisfaction in parenting. Cronbach’s alpha coefficients were α = 0.75 for efficacy and α = 0.73 for satisfaction. For both subscales, mean scores were computed, with higher scores indicating greater parental sense of competence.

*Parenting styles and dimensions questionnaire* [63]: This 32-item instrument consists of three scales measuring authoritarian, authoritative, and permissive parenting. The authoritative items reflect reasoning/induction, warmth and support, and democratic participation; the authoritarian items reflect verbal hostility, physical coercion, and nonreasoning/punitive strategies; and the permissive items reflect indulgence and failure to follow through. All items are answered on a five-point scale, with higher scores showing higher authoritative/authoritarian/permissive practices. Internal consistency in this study was α = 0.81 for authoritative practices, α = 0.79 for authoritarian practices, and α = 0.64 for permissive practices. Mean scores were computed.

*Parenting alliance inventory* [64]: This 20-item scale assesses the degree of commitment and cooperation between husband and wife in child rearing. For each item, parents respond on a 5-point scale. The total score revealed α = 0.94 in this study. We used the mean score, with higher scores indicating stronger support between partners as parents. 

*Family cohesion and adaptability scale* [65]. We used the FACES-III, which evaluates emotional bonding between family members, as well as the adaptability of the family system. It is ranked on a 5-point scale. Unlike other versions, the scores assessed with FACES-III are interpreted in a linear manner, so the higher the score, the greater the level of family cohesion and adaptability. Internal consistency in this research was α = 0.74 for cohesion and α = 0.56 for adaptability. Mean scores were computed.

### 2.4. Procedure

Mental health practitioners referred the families for SSFT intervention. SSFT practitioners enrolled the families in SSFT if they met the following criteria: (1) A child under 18 was being treated by the mental health service; (2) the referred child met ICD-10 criteria for: Pervasive developmental disorders; behavioral and emotional disorders with an onset usually occurring in childhood and adolescence; neurotic, stress-related, and somatoform disorders; and if the previous criteria were not met, the child had to meet the requisites for an eating disorder process or severe mental illness; and (3) SSFT practitioners, based on their professional criteria through the observation and interviews with both the adolescent and the parents, considered that the child’s symptomatology could be related with a family dysfunction (e.g., the symptomatology was limited to the family context, parental disagreement or dysfunctional communication patterns) or that the family dynamic was either being impacted by the symptomatology or maintaining it (e.g., difficulties in adjusting to changes due to adolescence or parental practices not coherent with the adolescent period, frequent or persistent family conflicts). If the intervention criteria were met, SSFT practitioners enrolled the family in the trial if they had an adolescent member (10 years or older).

Two trained researchers, external to the SSFT, interviewed the caregivers and practitioners of each family and assessed the adolescents at the mental health service facilities. The pre-test was completed before the first SSFT session, and the post-test in the last session (for those families that had attended at least three intervention sessions). The average length of time between pre- and post-test assessment was 10 months, which corresponded approximately to the school year. Every informant participated in the study voluntarily, after signing an informed consent form in accordance with the Declaration of Helsinki. The aims of the research project were explained, and all participants were assured that their anonymity would be protected. Ethics approval was obtained from the ethics committee of the Andalusian Health Services (code 22/0509). No monetary incentives were offered. 

**Figure 1:**
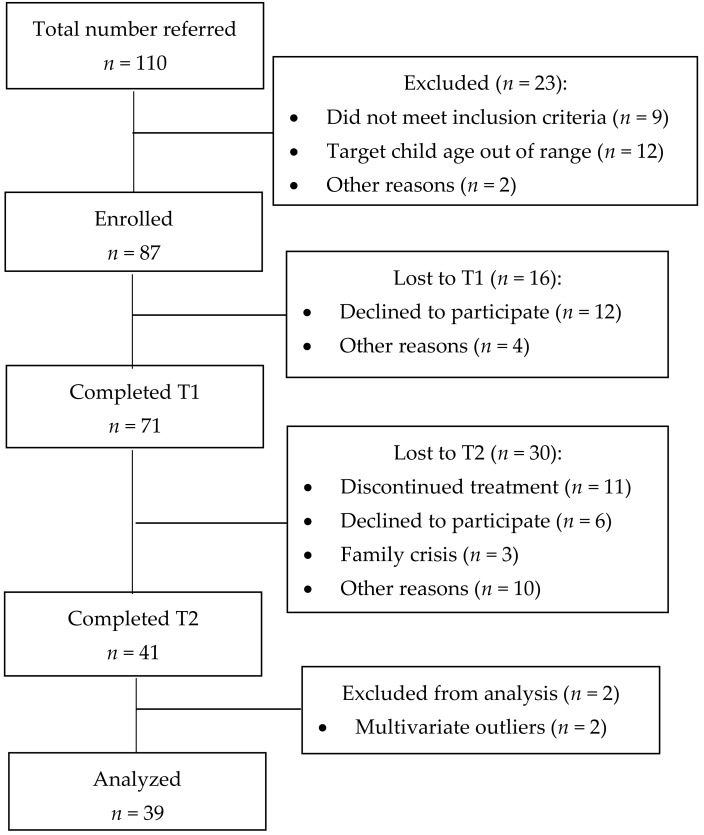
Flowchart of participants through the study.

The flow of cases through the trial is shown in Figure 1. Patients were classified as dropouts if they did not complete Time 2 assessment protocols, despite being contacted at least three times by the research team. The dropout rate at Time 2 was 42.25%. 

Dropouts and completers were compared in all pretreatment variables using one-way ANOVAs for quantitative variables and Chi-square tests for qualitative ones. Partial eta squared and Cramer’s V were computed as effect-size indices. Partial eta squared was considered small if <0.01, medium if ≥0.06 and <0.14, or large if >0.14; Cramer’s V was considered small if <0.30, medium if >0.30 and <0.50, or high if >0.50 [66]. Significant differences were not found in any variables, except for parenting alliance (see Table 1).

### 2.5. Data Analyses

Statistical analyses were performed with SPSS v-18 (SPSS Inc., Chicago, IL, USA) [67]. Missing data at item level were extrapolated using the missing value analysis. When more than 10% of the items from a questionnaire were missing, the case was excluded from the corresponding analysis. If this were not the case, we then applied the SEM procedure to impute the data, having previously checked that the data were missing at random using Little’s MCAR test. We found less than 5% of missing data with an MCAR distribution.

We examined univariate and multivariate outliers using box plots and Mahalanobis’ distance, respectively [68], finding two multivariate outliers which we excluded from subsequent analyses. Other statistical assumptions for parametric tests were checked and confirmed following Hair, Anderson, Tatham, and Black’s [69] recommendations (i.e., linearity, normality, homogeneity, and absence of multicollinearity and singularity). As an exception, high kurtosis for parental alliance required a reflected and logarithmic transformation. 

We based statistical conclusions on effect-size indices when statistical significance did not reach significance due to small sample size. We examined main and interaction effects from mixed factorial ANOVAs for the analyses of effectiveness, considering the pre-post measures as within the subjects’ factor (change) and informant’s gender as between the subjects’ factor. We used partial eta squared as an effect-size index, with the conventional limits of 0.01, 0.06, and 0.14 for the small, medium, and large levels of effect size, respectively [66].

## 3. Results

First of all, we examined the main effect of gender and found neither a significant effect nor a medium or large effect size. As Table 2 shows, after controlling for gender, the change between pre- and post-measures was significant for several dependent variables. Thus, the adolescents exhibited fewer internalizing and externalizing problems in the post-test with a high effect size. In turn, parents reported higher satisfaction, as well as fewer authoritarian and permissive practices, also with a high effect size. Moreover, higher efficacy as a parent and more authoritative practices were reported with a medium effect size. Finally, the interaction between change and gender was significant for the parenting alliance variable, with a high effect size.

**Figure 2:**
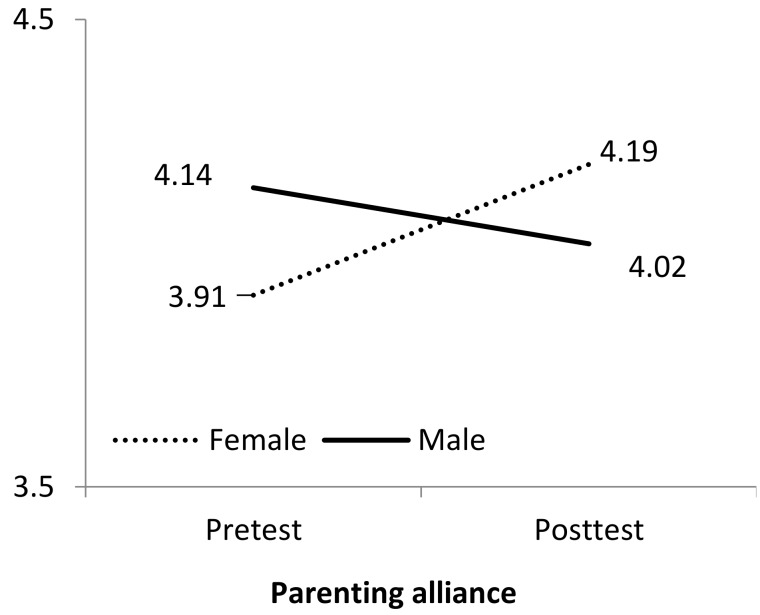
Interaction effect of gender on parenting alliance.

The change * gender interaction is plotted in Figure 2, and it shows that mothers improved their parenting alliance after intervention, while the opposite occurred with fathers. To investigate further into the interaction effect, we performed a simple repeated measures ANOVA for each gender. The results showed that mothers significantly improved their parenting alliance after treatment with a high effect size, *F*(1,18) = 4.54, *p* = 0.047, η^2^_partial_ = 0.20, but no statistical difference was observed for fathers, *F*(1,18) = 0.24, *p* = 0.628, η^2^_partial_ = 0.01.

## 4. Discussion

The results of this study have shown a positive impact of a structural–strategic oriented family therapy on both the parents and adolescents in the family, dyadic, and individual-level dimensions. The improvement observed after the intervention was independent of the gender of both parents and adolescents, barring the parenting alliance variable. 

The systemic approach understands the family as a whole, not as a simple sum of individual members. According to this approach, a common objective in structural family therapy, regardless of clients’ needs, consists of empowering and strengthening the family as a system, favoring the persistence of these changes over time [38]. In consonance with previous empirical evidence [35,36], this study shows the impact of this approach in the family sphere, particularly in terms of improving family cohesion. This result is particularly relevant with vulnerable families facing difficulties associated with the readjustment of family roles and norms, response to new demands and needs of family members [9,13,14]. This is the case of families with adolescents suffering from mental health problems, due to the existence of additional needs, demands, and difficulties linked to the presence of the mental disorders [15]. Nevertheless, despite the importance of the abovementioned results, no improvement was observed in family adaptability. Families in this situation tend to behave inflexibly when negotiating and learning new ways of resolving parent–adolescent conflict [42]. An improvement in family adaptability in this population would have been remarkable; the absence of changes in this dimension may be due to reliability problems when assessing with FACES [70].

At a dyadic level, authoritative parental practices increased after the treatment, and both authoritarian and permissive practices decreased. Only a handful of studies had previously assessed the effectiveness of a systemic family approach on families whose adolescents presented mental health problems in dyadic dimensions [44]. Parenting training in childrearing practices constitutes a core component of most family interventions, particularly when child behavior problems exist [38]. Parental practices based on affect, dialogue, and reasoning are related to better family functioning [71] and adolescent adjustment [6,72,73].

The structural–strategic therapy tested in this study has also shown other dyadic effects. Participant mothers reported feeling more support from fathers in childrearing, although the opposite was not found (fathers feeling more supported by mothers). This result is not surprising considering that mothers are usually involved more in childrearing than fathers and also feel less supported in their parenting role [59]. This difference in gender may also be explained because mothers reported a lower level of parenting alliance before the intervention, and therefore had greater scope for subsequent improvement compared to fathers. 

At an individual level, participating parents reported better parental sense of competence after the therapy. Thus, both fathers and mothers reported higher perceived efficacy and satisfaction as a parent. Again, this result is particularly relevant as parents from these families presented high levels of difficulty in exerting their parental role [15]. For example, there is evidence of the existence of additional parental stress on parents with adolescents presenting mental health problems, and the relationship between parental stress and less perceived efficacy and satisfaction as a parent [74]. Consequently, the increase observed in parental sense of competence could be mirrored by a decrease in parenting stress. In any event, the improvement in parental sense of competence is positive not just for parents at an individual level, but also for the adolescents and the family as a whole [53,75,76].

Finally, this study has shown positive results in adolescent behavior, regardless of gender [19,33,34]. The reduction in adolescent problematic behavior both at external and internal level confirms the usefulness of structural–strategic therapy. This result can be explained as a direct effect of the intervention or as an indirect effect of improvements in family functioning [35,36], parental practices [50,51,52], parental sense of competence [52,54], and parenting alliance [55]. As pointed out in the introduction, the absence of differences between boys and girls can be explained by the homogenization of adolescents’ daily experiences in today’s society [12].

This study has several limitations. First, a main shortcoming is the small sample of families recruited in the study. The high specialization and costs associated with SSFT together with the high-risk profile of these families help to understand this limitation. The latter, due to mental health problems and family dysfunction, can also explain the high dropout rate reported in this study. Whatever the reason is, the statistical strength of the study could be improved with a higher sample size, particularly if considering the statistical conditions of the longitudinal analyses [77]. Second, we would have liked to have been able to conduct a long-term analysis to examine the persistence of treatment effects in the mid to long term. Third, the most important limitation of this study was the absence of a comparison group to enable us to corroborate that changes between pre-test and post-test were due to the therapy and not to other circumstances [78].

## 5. Conclusions

Despite the abovementioned limitations, this study has made some contributions. We drew on previous findings about the effectiveness of family-oriented and family-based interventions with adolescents with mental health difficulties [19,20] from family systemic therapy approach [19,27,28,29,30,31]. While reaching the gold standard for effectiveness remains a distant goal for structural–strategic family therapy, this paper offers some evidences about its usefulness for improving individual, dyadic, and family adjustment in families with adolescents with mental health difficulties [39].

In sum, this study has practical implications concerning the way specialized services for children and adolescents with mental health problems have been traditionally organized, and regarding the core elements that need to be specifically targeted when working with these families. In general, specialized mental health services for children and adolescents have traditionally focused on symptom reduction and “parental training”, which have proven to be useful and essential interventions. However, our results support the importance of incorporating complementary approaches targeting families as a whole in their regular services as to adequately address the complex needs and difficulties of families of adolescents with mental health issues [23]. In addition, this study highlights the need to directly target certain core elements related to the dyadic parental relationship and the parent–child relationship when intervening with families of adolescents with mental health problems. Finally, gender-related results support the idea of differentiated approaches when working at a dyadic parental level, such as co-parenting. Mothers and fathers seem to not only experience co-parenting differently but also respond differently to interventions that directly target this core element [59]. Therefore, this study highlights the relevance of taking into account and incorporating gender-based strategies in interventions.

## Figures and Tables

**Table 1:** Baseline characteristics for completers and dropouts.

	Completers %/M	Dropouts %/M	Differences χ^2^/F
**Target adolescent**			
Girls	73.17%	56.67%	2.11
Age	14.12	14.14	0.01
**Family**			
No. of family members	3.82	4.04	0.83
No. of children	1.80	1.60	1.86
Two-parent structure	90.24%	81.48%	1.09
**Behavior problems**			
Internalizing	0.50	0.52	0.04
Externalizing	0.55	0.56	0.01
**Parental competence**			
Efficacy	3.10	3.22	0.26
Satisfaction	3.77	3.88	0.32
**Parental practices**			
Authoritative	3.65	3.67	0.02
Authoritarian	1.84	1.83	0.02
Permissive	2.35	2.54	1.09
**Parenting alliance**	4.03	3.59	5.21* η^2^_partial_ = 0.08
**Family functioning**			
Cohesion	3.65	3.44	2.00
Adaptability	2.64	2.76	1.16

*p < 0.05.

**Table 2:** Descriptives and inferential statistics for change and change * gender interaction of the mixed factorial ANOVAs for each dependent variable.

	Descriptives M (SD)		Change F (η^2^_partial_)	Change × Gender F (η^2^_partial_)
	Pre-Test	Post-Test		
**Behavior problems**				
Internalizing	0.48 (0.21)	0.33 (0.19)	14.74*** (0.38)	0.02 (<0.01)
Externalizing	0.55 (0.26)	0.35 (0.21)	20.72*** (0.46)	0.47 (0.02)
**Parental competence**				
Efficacy	3.14 (0.68)	3.32 (0.64)	4.04* (0.10)	0.88 (0.02)
Satisfaction	3.76 (0.70)	3.98 (0.81)	5.19* (0.14)	0.12 (<0.01)
**Parental practices**				
Authoritative	3.61 (0.50)	3.75 (0.53)	4.25* (0.11)	0.21 (0.01)
Authoritarian	1.84 (0.46)	1.65 (0.40)	11.30** (0.25)	0.23 (0.01)
Permissive	2.31 (0.77)	2.05 (0.56)	5.44* (0.14)	2.08 (0.05)
**Parenting alliance**	4.03 (054)	4.11 (0.63)	0.89 (0.02)	**2.94 (0.08)**
**Family functioning**				
Cohesion	3.62 (0.44)	3.73 (0.45)	**3.26 (0.08)**	0.13 (<0.01)
Adaptability	2.65 (0.42)	2.73 (0.44)	0.91 (0.03)	0.39 (0.01)

*Note*. Boldfaced contrasts indicate medium or high effect sizes. * *p* < 0.05, ** *p* < 0.005, *** *p* < 0.001.
